# Role of thalidomide in angiodysplasia-related gastrointestinal bleeding: a systematic review

**DOI:** 10.3389/fgstr.2026.1669563

**Published:** 2026-02-26

**Authors:** Junaid Khan, Amna Yousaf Shah, Aamir Asif Khan, Ruqaiya Shahid Raja, Zubair Ahmed, Mahad Shahid Raja, Simona Eng, Qamar Iqbal

**Affiliations:** 1 Department of Internal Medicine, Tidal Health Peninsula Regional, Salisbury, MD, United States; 2 Department of Internal Medicine, University at Buffalo, Buffalo, NY, United States; 3 University Hospital, Kerry, Ireland; 4 Shifa College of Medicine, Islamabad, Pakistan; 5 Fatima Memorial Hospital, Lahore, Pakistan; 6 Akhtar Saeed Medical College, Rawalpindi, Pakistan

**Keywords:** angiodysplasia, arteriovenous malformation, gastrointestinal bleed, GIB, thalidomide

## Abstract

**Objective:**

Angiodysplasia of the gastrointestinal (GI) tract is a leading cause of occult GI bleeding, and its management remains challenging. Various pharmacologic and endoscopic therapies are used with limited success. This systematic review evaluates the clinical efficacy of thalidomide in angiodysplasia-related GI bleeding.

**Methods:**

A comprehensive literature search was conducted across PubMed, Embase, Scopus, and CINAHL using MeSH terms “Vascular malformations” OR “Angiodysplasia” AND “Thalidomide,” covering the period from 1994 to February 16, 2024. We included clinical trials and case series with at least five adult patients treated with thalidomide for angiodysplasia-related GI bleeding. Six studies met the inclusion criteria: two randomized controlled trials (RCTs), one retrospective observational study, and three case series.

**Results:**

A total of 265 patients were included, with a median age of 63.5 years; 37% were male. Angiodysplasia was diagnosed using endoscopy, colonoscopy, or push enteroscopy. Clinical outcomes varied across studies. Garrido et al. reported an 84% response rate based on hemoglobin improvement. In an RCT, Chen et al. demonstrated reduced bleeding episodes in 68.6% of patients receiving thalidomide 100 mg compared with 51% in the 50 mg group. Ge et al. reported a response rate of 71.4% (20/28) in the thalidomide group versus 3.7% (1/27) in the iron group (risk difference 67.7%, 95% CI 51.1–84.2). Common adverse effects included constipation, dizziness, fatigue, limb numbness, and peripheral neuropathy.

**Conclusion:**

Thalidomide appears effective in reducing bleeding episodes in angiodysplasia-related GI bleeding. However, heterogeneity in dosing, outcome definitions, and safety reporting highlights the need for larger, standardized trials to clarify optimal treatment strategies and long-term safety.

## 1 Introduction

Angiodysplasia is a critical challenge in vascular health, characterized by abnormal connections between arteries and veins resulting from the failure of the embryonic vascular plexus to form mature capillary beds (1, 2). When present in the GI tract, angiodysplasia can manifest as mild iron deficiency anemia or melena, and in severe cases, it can lead to alarming rectal bleeding. The region most frequently impacted includes the cecum and right colon, followed by the jejunum, ileum, and duodenum (4). Angiodysplasia is a leading cause of occult gastrointestinal bleeding among the elderly population. Real-world endoscopic cohorts show that gastrointestinal angiodysplasia predominantly affects older patients and carries a substantial bleeding risk, with approximately one-quarter of patients experiencing bleeding; anticoagulant use, particularly warfarin, is the strongest independent predictor of hemorrhage (5) (41). Despite the availability of various treatment options—surgical, endoscopic, and pharmacological—success rates remain unsatisfactory. The lack of FDA-approved therapies reveals a limited understanding of this complex condition but also raises concerns about the increased risk of recurrent bleeding and potential complications. Effective surgical outcomes depend heavily on selecting favorable patient profiles, whereas endoscopic interventions often fall short, particularly when multiple challenging anatomical sites are involved (3, 6). Gastrointestinal angiodysplasia is believed to develop as a result of chronic or intermittent mucosal hypoperfusion, leading to localized hypoxia and, consequently, activation of angiogenic signaling pathways. Hypoxia drives angiogenesis through the induction of hypoxia-inducible factors and downstream mediators like vascular endothelial growth factor (VEGF) (16). Studies of human tissue have shown increased expression of angiogenic factors in colonic angiodysplasia compared to normal mucosa (17). Studies further report that unregulated vascular remodeling and impaired vessel maturation are central features of gastrointestinal angiodysplasia (28). In this systematic review, thalidomide-treated patients were predominantly older with significant comorbidity; inconsistent reporting of anticoagulant use limits adjustment for baseline bleeding risk and may contribute to inter-study variability in treatment response (41). Our review highlights the potential of thalidomide in addressing angiodysplasia-related gastrointestinal bleeding, underscoring the need for comprehensive research in this area. By investing in further studies, we can enhance our understanding and management of angiodysplasia, ultimately improving outcomes for patients and reducing the burden of this condition on healthcare systems.

## 2 Methods

### 2.1 Literature search

We conducted a literature search across four databases (PubMed, Embase, Scopus, and CINAHL) using the MeSH terms ““Vascular malformations” OR “Angiodysplasia” AND “Thalidomide,” covering the period from 1994 to February 16, 2024. Two independent authors performed an initial and subsequent screening of the search results by the Preferred Reporting Items for Systematic Reviews and Meta-Analyses (PRISMA) guidelines. This review was not registered in PROSPERO; this is acknowledged as a methodological limitation.

### 2.2 Inclusion and exclusion criteria

Our inclusion criteria included adults aged 18 years or more who were diagnosed with angiodysplasia and were only treated with thalidomide. We included RCTs, observational studies, and only those case series that included more than 5 patients. Exclusion criteria included treatment of angiodysplasia with any other modality, either pharmacological or surgical, case reports, case series with less than 5 cases, Studies not published in English, or those without accessible full‐text versions were also excluded. Studies without clear outcome measures and literature review were also excluded. Studies exclusively focused on gastric antral vascular ectasia (GAVE) were excluded to maintain a homogeneous angiodysplasia population.

### 2.3 Data extraction

**Figure 1:**
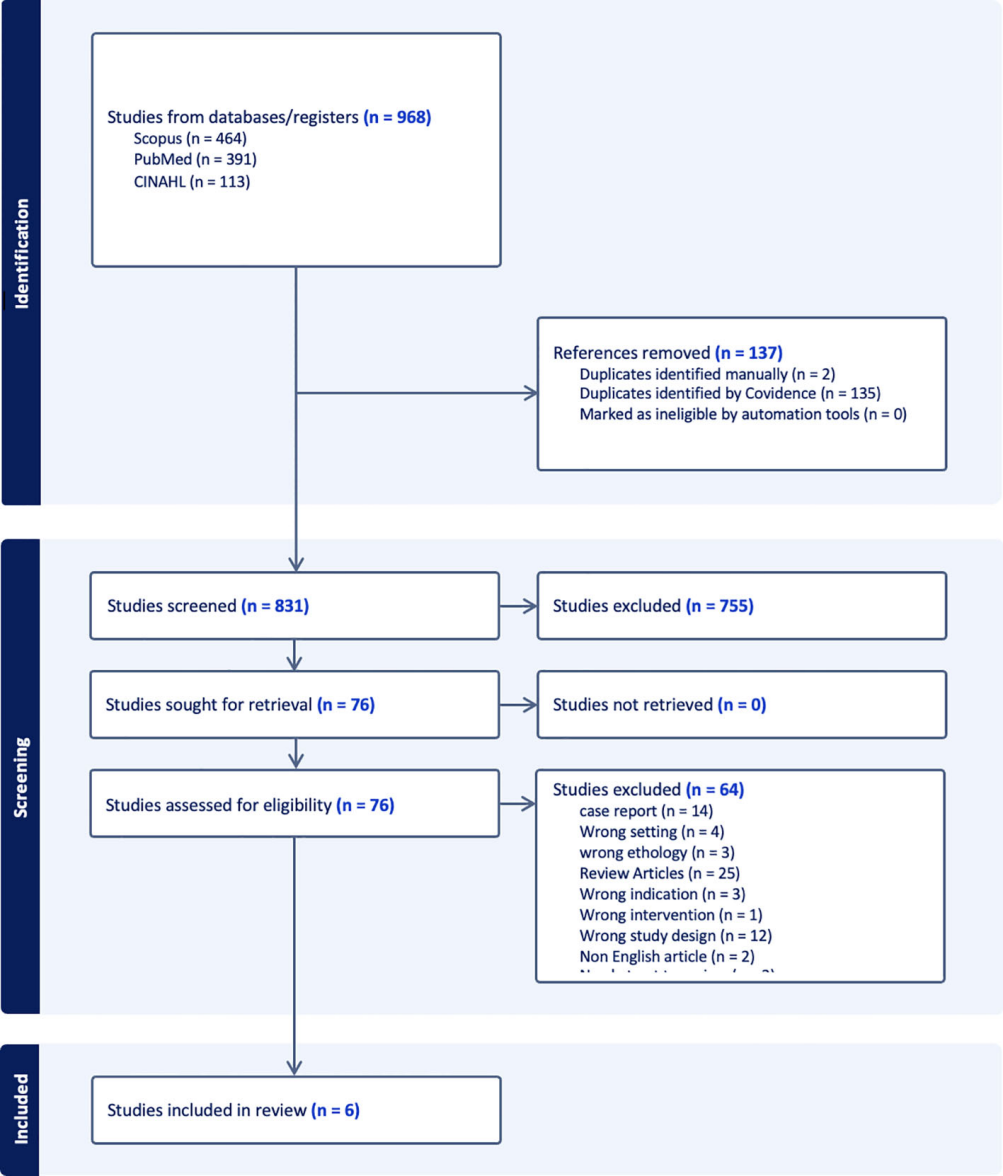
PRISMA flow diagram.

We included clinical trials and case series of five or more adult participants treated with thalidomide for AVM-related GI bleeding. For our data generation and analysis, we identified six studies, including two prospective randomized controlled trials (one of which was open-label), one retrospective observational study, and three case series (each involving more than five cases) (7–11, 36) (See **Figure 1**).

### 2.4 Quality assessment

Quantitative meta-analysis was not performed due to substantial clinical and methodological heterogeneity across included studies. In particular, response was variably defined using distinct outcome constructs, including hemoglobin rise, reduction in transfusion requirements, and ≥50% reduction in bleeding episodes, which are not directly comparable measures of treatment effect. Additional sources of heterogeneity included differences in thalidomide dosing regimens, follow-up duration, study design (RCTs vs observational series), and patient populations (e.g., LVAD-associated bleeding). Pooling these disparate endpoints into a single quantitative estimate would risk producing misleading summary effects. Accordingly, results were synthesized narratively, with outcomes interpreted within the context of their original definitions. We used the Cochrane Risk of Bias version 2 (RoB2) tool for randomized controlled trials, the Risk of Bias in Non-Randomized Studies of Interventions (ROBINS-I) tool for the prospective observational study, and the Joanna Briggs Institute (JBI) checklist for case series. For RoB2 domains 1-5, all signaling questions were answered using standard response options: yes, probably yes, probably no, no, and no information. Overall judgments followed the decision algorithms for each tool (7–11, 36). All appraisals were discussed by two independent researchers (RSR, MSR), and the final decision was made by consensus, with a third reviewer available for arbitration. Detailed responses to the signaling questions are provided in the **Supplementary file S1**, and domain-level summaries are presented in **Supplementary tables S1**-**S3**.

## 3 Results

**Figure 2:**
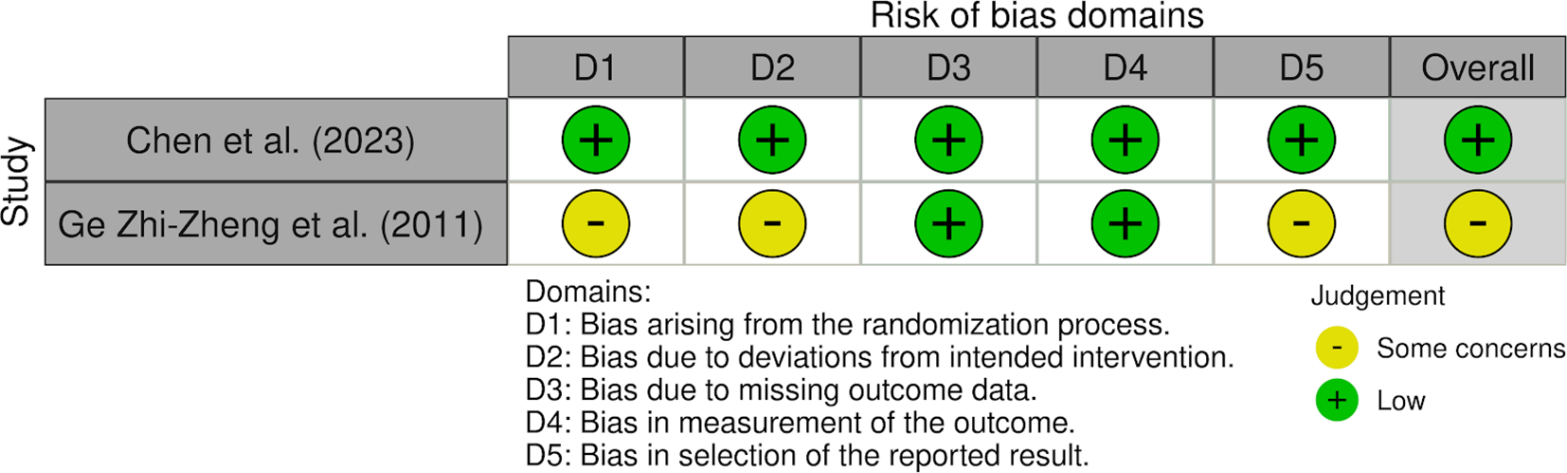
Traffic light plot for RoB 2 studies. Traffic-light plot of risk of bias for randomized controlled trials, Chen et al. (2023) and Ge Zhi-Zheng et al. (2011) which was assessed using the Cochrane Risk of Bias version 2 tool. Each domain is color coded to its corresponding judgement as presented in **Supplementary table S2**. Green is for low risk of bias, yellow is for some concerns and red is for high risk of bias.

**Figure 3:**
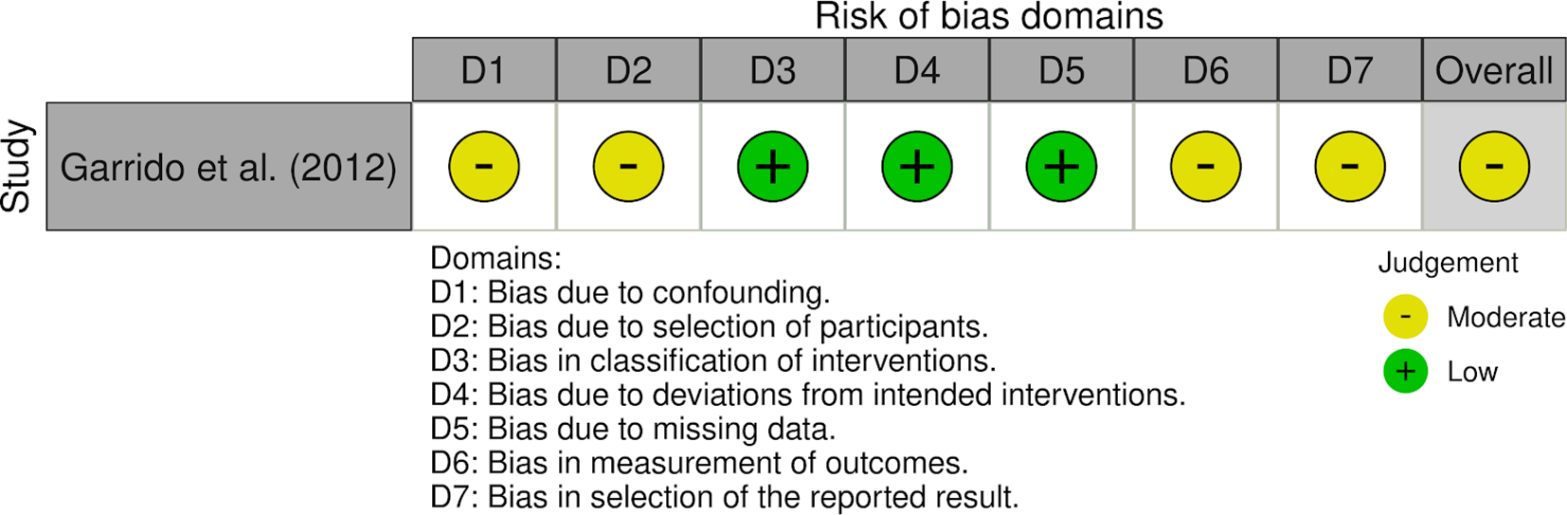
Traffic light plot for ROBINS-I study. Traffic-light plot for risk of bias for observational study, Garrido et al. (2012) which was assessed using the Cochrane Risk of Bias in Non-Randomized Studies of Intervention (ROBINS-I) tool. Each domain is color-coded and their judgements correspond to the **Supplementary table S3**. Green is for low risk of bias, yellow is for moderate risk of bias and red is for high risk of bias.

We analyzed six studies on the treatment outcomes of thalidomide for AVM-related GI bleeding (7–11). A total of 265 participants were included, with a median age of 63.5 years, and 37% (n=100) were male. AVMs were diagnosed using endoscopy, colonoscopy, or push enteroscopy. Study characteristics are described in (**Table 1**), and primary endpoints are defined in **Table 2**. Garrido et al. reported the highest response rate of 84.60%, as indicated by improvement in hemoglobin levels (10). Draper et al. (2015) was included as a case-series subgroup because it provides unique safety and efficacy data in LVAD-associated angiodysplasia, despite its small sample size (9). In a randomized trial, Chen et al. found that 68.6% (n=35/51) of participants receiving 100 mg of thalidomide experienced fewer bleeding episodes. In comparison, 51% (n=25/49) of those treated with 50 mg of thalidomide also reported decreased bleeding episodes compared with 16% (n=8/50) in the placebo group (the risk difference 52.6%, 95% CI 35.0-70.3; P < 0.001) (8). Likewise, Ge et al. showed a significant improvement, with 71.4% (n=20/28) of patients in the thalidomide group having a ≥50% reduction in bleeding episodes compared with 3.7% (n=1/27) in the iron control group (risk difference 67.7%, 95% CI 51.1-84.2; P < 0.001) (11). The adverse effects that mostly occurred with the use of thalidomide, as mentioned in Chen et al. were constipation in 21% (n=32/150), limb numbness in 11% (n=17/150), and dizziness in 6% (n=9/150), Dose-related increases were observed as higher in 100mg of thalidomide as compared to 50mg (8). Ge et al. also mention adverse events such as fatigue in 11% (n=6/55) and peripheral neuropathy in 5% (n=3/55) of patients (11). Most studies noted common side effects, including fatigue, constipation, rash, and neuropathy. These side effects tend to improve with either a dose reduction or the cessation of medication. The double blind randomized controlled trial by Chen et al. received an overall low risk of bias across all domains in the RoB 2 tool. Randomization produced well-balanced groups and blinding ensured minimal deviations and selection bias; missing outcome data were minimal, and the study followed the intention-to-treat protocol (8). In contrast, the open-label randomized controlled trial by Ge et al. was judged to have some concerns, mostly due to unclear allocation concealment, lack of blinding, and a prespecified protocol; however, missing data and outcome measurement domains were judged at low risk (11). The prospective observational study by Garrido et al. was judged at moderate risk of bias using the Risk of Bias in Non-randomized Studies of Interventions (ROBINS-I) tool, with the main limitations being confounding, selection bias, outcome measurement, and selection of reported results despite complete follow-up and absence of bias in interventions (10). The case series included most of the Joanna Briggs Institute (JBI) checklist items, but were judged as moderate in quality because of reasons such as a small sample size and unclear consecutive inclusion of participants (7, 9, 36). The overall results of all included studies and the quality assessment tools are summarized in **Table 3**. Domain-level judgements for RoB 2 and ROBIN-I tools are available as traffic light plots in **Figure 2** and **Figure 3**, respectively. The results of the assessment are summarized in a table (**Table 1**).

**Table 1:** Overview of included studies.

Author(s) year	Study type	Number of patients	Age years (mean)	Male: female ratio n (%)	Cause of GIB*	Prior failed treatments	Thalidomide dose (mg)		Thalidomide duration (months)	Primary outcome	Follow up months	Loss to Follow up	Average Hb prior to treatment g/dl	Average Hb after treatment g/dl	Response rate* (%)	
Chen Huimin et al. (2023)	Double blind RCT	150	47.5	61:89 (40:59)	GIAD: 150	NA	100 n: 51	50 n: 49	4	≥50% reduction in bleeding episodes as compared to last year	12	Minimal (<5%)	NA	NA	100mg 35/51 (68.6)	50mg 25/49 (51.0)
Zhi-Zheng et al. (2011)	RCT (prospective, open-label)	55	63.5	23:32 (42:58)	GIAD: 80 (100)	EGD Colonoscopy	100		4	≥50% reduction in bleeding episodes as compared to observation year	12	Minimal	6.4	10.2	43/55 (78.2)	
Garrido Antonio et al. (2012)	Prospective observational study	12	77	7: 5 (58:42)	NA	Octreotide Endoscopy	200		4	Decrease in transfusions and bleeding episodes	2	None reported	6.5	12.1	11/12 (91.7)	
Bayudan Alexis et al. (2020)	Retrospective case series	15	69	9: 6 (60:40)	GIAD: 10 (66) Non GIAD: 5 (33)	Octreotide: 6 (40%) Estrogen: 1 (6%) Aminocaproic acid: 1 (6%)	Minimum: 50 Maximum: 200		28	Recurrence of GIB after 6 months on Thalidomide	45	Not reported	7.9	NA	5/13 (38.5)	
Draper et al. (2015)	Case series	8	63	NA	GIAD: 6 (75) Non-GIAD: 2 (25)	NA	Minimum: 50 Maximum: 200		6	Bleeding Cessation	NA	Not reported	NA	NA	6/8 (75.0)	
Kamalaporn et al. (2009)	Open-label prospective case series	7	NA	NA	GIAD	endoscopic therapy, blood transfusions	100-200mg/day (dose escalation)		6	Reduction in transfusion requirement and bleeding episodes	6	4/7 (57.1%)	NA	NA	3/7 (42.9)	

*Ge, Zhi-Zheng et al. and Chen Huimin et al., 2023; defined response rate as > 50% decrease in bleeding events in 1st year treatment.

*Garrido, Antonio et al.; defined response rate as mean Hgb improvement after 4 months of treatment.

*Bayudan Alexis et al.; defined response rate as reduction in number of blood transfusions.

*Draper et al.; defined response rate as decrease in number of bleeding episodes.

*Kamalaporm et al.: defined response rate as reduction in transfusion requirement and bleeding episodes.

GIB: gastrointestinal bleed, GIAD: gastrointestinal angiodysplasia.

**Table 2:** Outcomes definition.

Author(s) year	Primary endpoint	Outcome definition
Chen Huimin et al. (2023)	≥50% reduction in bleeding episodes as compared to last year	Reduction in bleeding 1 year after the treatment compared with number that occurred during observation period
Zhi-Zheng et al. (2011)	≥50% reduction in bleeding episodes as compared to observation year	Reduction in bleeding 1 year after the treatment compared with number that occurred during observation period
Garrido Antonio et al. (2012)	Decrease in transfusions and bleeding episodes	Improvement in hemoglobin from baseline to 4 months
Bayudan Alexis et al. (2020)	Recurrence of GIB after 6 months on Thalidomide	Decrease in recurrence of Gastrointestinal bleed, hospitalization, blood transfusion and endoscopic therapies.
Draper et al. (2015)	Bleeding Cessation	No recurrent transfusion-requiring bleeds
Kamalaporn et al. (2009)	Reduction in transfusion requirement and bleeding episodes	No RBC transfusions during 6-month therapy

**Table 3:** Quality assessment of studies.

Study	Tool used	Assessment
Chen et al. (2023) (8)	RoB 2	Low risk of bias
Zhi-Zheng et al. (2011) (11)	RoB 2	Some concerns of bias
Bayudan et al. (2020) (7)	JBI	Moderate quality
Draper et al. (2015) (9)	JBI	Moderate quality
Kamalaporn et al. (2009) (36)	JBI	Moderate quality
Garrido et al. (2012) (10)	ROBINS-I	Moderate risk of bias

## 4 Discussion

Angiodysplasia can be isolated or associated with syndromes such as hereditary hemorrhagic telangiectasias (HHT) (12). In 88% of patients, angiodysplasia is asymptomatic and is often an incidental finding. However, when symptomatic, symptoms are consistent with their anatomic location (13).

Although gastric AVMs are rare, they are often found in the colon (especially the cecum and ascending colon). Angiodysplasia makes up for 5% of total upper GI bleeds, while 30% of GI bleeds in the elderly are secondary to angiodysplasia (14). Diagnoses are challenging, as these lesions are often missed on routine endoscopy; therefore, their presentation is classified as obscure gastrointestinal bleeding (15). By nature, angiodysplasia involving the GI tract are intermittently symptomatic, oftentimes presenting as iron deficiency anemia, coinciding with occult blood loss (3).

To this day, the exact pathophysiology of angiodysplasia remains unclear. Recurrent bleeding in gastrointestinal angiodysplasia is attributed to dilated, structurally fragile vascular channels that are prone to rupture. A recent hypothesis is that angiodysplasia arises from chronic intermittent obstruction of vessels, which leads to local hypoxia and, in turn, the expression of multiple neurovascular growth factors, thereby promoting the formation of new blood vessels (16, 17). As a result, several treatment modalities targeting angiogenic signaling pathways have been explored in limited clinical studies, with mixed results. Other treatments include octreotide (18), endoscopic coagulation (19), selective embolization, hormonal therapy (20), and surgical resection (21). Surgical resection is often the last resort because of high perioperative morbidity (23%) and mortality rates (12%) (21).

Thalidomide’s efficacy in this setting is supported by its anti-angiogenic and immunomodulatory properties. Experimental studies using human intestinal microvascular endothelial cells have shown that thalidomide inhibits TNF-α-mediated NF-κB activation and suppresses angiogenic responses, resulting in decreased vascular proliferation and bleeding risk (38). Thalidomide has also been shown to downregulate VEGF-mediated angiogenesis and to modulate inflammatory cytokine signaling, thereby stabilizing vascular lesions (6, 39). However, these effects are associated with dose- and duration-dependent adverse effects, such as peripheral neuropathy, highlighting the need for careful patient selection and close monitoring (37, 39).

These mechanistic and safety considerations have been comprehensively synthesized in a narrative review by Bauditz, which emphasizes that clinically relevant adverse effects, such as peripheral neuropathy and sedation, are largely dose- and duration-dependent and can often be mitigated through careful patient selection, dose adjustment, and close monitoring (42). Given the predominantly elderly population affected by angiodysplasia, teratogenic risk is of limited relevance, whereas neurologic and thromboembolic surveillance remain essential. This narrative synthesis supports both the biologic plausibility and cautious clinical applicability of thalidomide in refractory angiodysplasia-related gastrointestinal bleeding (42).

Although hormonal therapy with estrogen and progesterone can help minimize bleeding, its routine use remains controversial and is generally discouraged due to conflicting evidence regarding its efficacy (20, 22, 23). Notably, because of their hypercoagulability, these agents are contraindicated in individuals with a history of thrombotic events or coagulation disorders (24).

Endoscopic coagulation techniques include argon plasma coagulation (APC), bipolar coagulation, and hemoclip placement (19). The re-bleeding rate was 7% higher than in patients who received no treatment. Moreover, the findings depend heavily on the endoscopic techniques used, patient comorbidities, lesion distribution, and follow-up time (25). Nevertheless, endoscopic interventions are considered invasive and are reserved for second-line treatment (26).

Non-invasive treatment modalities, such as octreotide (a somatostatin analog), are often used to manage bleeding episodes (18). The feasibility of this treatment lies in its ability to be administered by injection, either subcutaneously or intramuscularly. Octreotide acts on vascular endothelial growth factor (VEGF) and modulates hemodynamics in the splanchnic circulation (27, 28). It increases vascular resistance, inhibits pepsin and gastrin secretion, and enhances platelet aggregation (29). Pertinent side effects include early abdominal pain, skin rash, and pain at the injection site (3), sinus bradycardia/arrhythmias, cholelithiasis, and biliary sludge (29).

Similarly, anti-angiogenic drugs such as bevacizumab, a monoclonal antibody that binds to VEGF and inhibits its activity, are often used to treat recurrent epistaxis in HHT (16) and have shown promising results in lung AVMs (30), hepatic AVMs (31), and intestinal AVMs (32). They are particularly useful in settings where bleeding is severe and unresponsive to treatment (33). In a study of bevacizumab, all three participants reported reductions in symptoms such as pain, bleeding, and pulsations, with no side effects, and one participant reported a visible decrease in lesion size (34).

A detailed review of the literature underscores the complexity of managing angiodysplasia, especially in patients with recurrent bleeding (35). Our systematic review assessed the use and efficacy of thalidomide for the management of angiodysplasia-associated gastrointestinal bleeding. Thalidomide is known to be effective in treating various diseases, including erythema nodosum, multiple myeloma, Behçet’s disease, graft-versus-host disease, and Crohn’s disease (21). Due to its anti-angiogenic properties and its ability to reduce VEGF expression, thalidomide can help control recurrent bleeding (21).

Substantial heterogeneity in outcome definitions was observed across studies evaluating thalidomide for gastrointestinal angiodysplasia–related bleeding. Kamalaporn et al. and Draper et al. (9, 36) focused primarily on transfusion elimination, while Garrido et al. assessed hematologic recovery using serial hemoglobin measurements (10). Bayudan et al. further expanded the outcomes to include hospitalizations and the need for endoscopic therapy (7). These endpoints capture distinct domains, such as biologic bleeding activity, healthcare utilization, and hematologic response, and are not interchangeable. As a result, direct quantitative pooling across studies is methodologically limited. Hemoglobin-based endpoints may be confounded by iron supplementation and transfusion practices, while transfusion-based outcomes depend on institutional thresholds. Bleeding-episode–based endpoints most directly reflect disease activity but require structured patient reporting. This outcome heterogeneity must therefore be considered when interpreting effect sizes and limits the certainty of comparative efficacy estimates.

Administration of thalidomide led to a reduction in bleeding episodes and a decreased need for transfusions, although response rates varied across the studies reviewed. The highest response rate, 84.6%, was reported by Garrido et al. (10) Four other studies also reported a lower mean transfusion volume in their thalidomide group (7–9, 11). Reduction in recurrent bleeding was observed with both high (200 mg/day) and low (50 mg/day) doses of thalidomide, with lower risks of side effects. Higher dosing was associated with greater toxicity and a higher incidence of adverse effects, such as peripheral neuropathy, which was reversible with early detection and cessation of therapy, and liver damage with mild to moderate elevation of AST and ALT (21). In contrast, lower thalidomide doses demonstrated reduced response rates but fewer side effects, as noted by Chen et al. (8). Kamalaporn et al. reported that 57% of patients (4 out of 7) discontinued thalidomide due to side effects, highlighting challenges to adherence. The side effects mainly include fatigue, peripheral neuropathy, and severe urticarial rash (36). Thalidomide also causes a dose-dependent, length-dependent axonal neuropathy that is irreversible in 25-75% of patients despite drug discontinuation, with recovery taking 4–6 years when it occurs (43, 45). The neuropathy exhibits a “coasting effect”—progression for weeks to months after stopping the drug—and symptoms may resolve slowly or not at all (43, 44). Daily dose is the most critical risk factor, with severe neuropathy now below 10% using doses ≤200 mg/day for <18 months, but immediate discontinuation at first symptoms is essential to limit irreversible axonal damage (43, 45). Adverse effects are summarized in **Table 4**.

**Table 4:** Thalidomide reported adverse effects.

Author(s)year	No of patients	Neuropathy (%)	Constipation (%)	Fatigue (%)	Rash (%)	Discontinuation due to toxicity (%)
Chen Huimin et al. (2023)	100	14/100 (14)	24/100 (24)	7/100 (7)	4/100 (4)	NA
Zhi-Zheng et al. (2011)	26	NA	6/26 (23)	9/26 (34)	1/26 (3.8)	2/26 (7.6)
Garrido Antonio et al. (2012)	12	1/12 (8)	NA	NA	NA	2/12 (16)
Bayudan Alexis et al. (2020)	13	2/13 (15)	1/13 (7.6)	3/13 (23)	NA	NA
Draper et al. (2015)	8	NA	NA	NA	NA	NA
Kamalaporn et al. (2009)	7	1/7 (14)	NA	2/7 (28)	1/7 (14)	4/7 (57)

*Percentages refer to only those who reported toxicity.

One major drawback of thalidomide is its teratogenicity; when used in pregnancy, it results in limb defects (10). The teratogenic effects are seen more often in the upper limbs (phocomelia, amelia, and polydactyly) and the face (facial palsy, small jaws, cleft lip, cleft palate), eyes (microphthalmia, anophthalmos, and poor vision), genitalia, and internal organs such as the heart, kidneys, and gastrointestinal tract (37). Thalidomide use is restricted under a mandatory risk evaluation and mitigation strategy (REMS) program with strict pregnancy prevention, controlled dispensing, and patient–prescriber agreements. Ongoing monitoring includes neurologic assessment, complete blood counts, and thromboembolic risk management throughout therapy (40).

## 5 Conclusion

The limited availability of published clinical studies and the variety of treatment approaches highlight our incomplete understanding of angiodysplasia and its management. This underscores the need for further research to develop effective management guidelines for this complex condition and to enhance clinical outcomes. Although data are limited, they are promising. Our review of the literature has shown that, when used appropriately, thalidomide could be beneficial in reducing the incidence of recurrent gastrointestinal bleeding from angiodysplasia. Further research is needed to clarify dosing and side effects when administering thalidomide.

## 6 Limitations

There are several limitations to our systematic review. First, the number of randomized controlled trials is limited. The included studies also differed in design, patient population, dosing regimens, response assessment, and follow-up duration, making direct comparisons difficult. None of the included studies systematically assessed molecular biomarkers such as VEGF or endothelial activation markers, limiting the ability to correlate molecular pathways, dose-response relationships, and treatment outcomes. Lastly, the review was not registered in PROSPERO, non-registration of the review protocol limits transparency and introduces the potential for selective outcome reporting. In a field characterized by heterogeneous outcome definitions and small sample sizes, this may influence the interpretation of treatment effects. Only English-language studies were included, which is acknowledged as a potential limitation.

## Data Availability

The original contributions presented in the study are included in the article/**Supplementary Material**. Further inquiries can be directed to the corresponding author.
