# Supplementary material for: Role of thalidomide in angiodysplasia-related gastrointestinal bleeding: a systematic review
Source: Front Gastroenterol (Lausanne). 2026 Feb 26;5:1669563. doi: 10.3389/fgstr.2026.1669563 (PMC12952340; doi:10.3389/fgstr.2026.1669563)
Supplement: Supplementary file 1 [file DataSheet1.docx]

**SUPPLEMENTARY MATERIAL**

| **Criteria** | **Bayudan et al. (2020)** | **Justification** | **Draper et al. (2015)** | **Justification** | **Kamalaporn et al. (2009)** | **Justification** |
| --- | --- | --- | --- | --- | --- | --- |
| Were there clear criteria for inclusion in the case series? | Yes | the criteria was clearly mentioned in the series, it included patients with refractory GI bleeding due to angiodysplasia or GAVE with comorbidities | Yes | the inclusion criteria was clearly mentioned as LVAD patients with refractory GI bleeding from angiodysplasia. | Yes | the inclusion criteria was clear it included patients who were transfusion-dependent chronic bleeding from angiodysplasias. |
| Was the condition measured in a standard, reliable way for all participants included in the case series? | Yes | confirmed by history and endoscopic evaluation | Yes | Consistent use of endoscopy and clinical data | Yes | clinical diagnosis and transfusion requirement monitoring |
| Were valid methods used for identification of the condition for all the participants included in the case series? | Yes | endoscopy (same method used for everyone) | Yes | endoscopic results and transfusion data | yes | endoscopic diagnostic methods |
| Did the case series have consecutive inclusion of participants? | Unclear | study does not mention if patients were chosen consecutively | Unclear | Consecutive inclusion not reported | Unclear | not explicitly stated |
| Did the case series have complete inclusion of participants? | Yes | All eligible patients were included | Yes | All patients treated in the centre during the study period. | Unclear | not explicitly stated |
| Was there clear reporting of demographics of the participants in the study? | Yes | Age, sex and other comorbids were clearly mentioned | Yes | Yes they were described clearly in the study | Unclear | limited baseline details reported |
| Was there clear reporting of clinical information of the participants? | Yes | They were reported clearly | Yes | They were reported | Yes | Reported clearly |
| Were the outcomes or follow up results of cases clearly reported? | Yes | the response rates to thalidomide and a reduction in transfusion rates was described | Yes | Response rates, bleeding episodes and transfusion history were reported and patients were followed throughout their treatment. | Yes | Response rates, transfusion pre/post discontinuation, side effects, 4/7 discontinued early and their reasons were reported |
| Was there clear reporting of the presenting site(s)/clinic(s) demographic information? | Yes | It was mentioned | Yes | The presenting site was mentioned | Yes | It was mentioned |
| Was statistical analysis appropriate? | Unclear | Due to the small sample size there was no advanced statistical analysis used | No | No statistical analysis was used. | No | It was descriptive only |
| **Overall appraisal** | **Moderate quality** | Major limitation is unclear consecutive inclusion and lack of statistical analysis but otherwise adequate | **Moderate quality** | Due to the small sample size a statistical analysis might not have been used, there was no clear consecutive inclusion of participants | **Moderate** **quality** | Due to small sample size, non-randomized, unclear consecutive recruitment, high discontinuation |

**Supplementary Table S1**

*The table includes the quality assessment for the case series, Bayudan et al. (2020) and Draper et al. (2015) using the Joanna Briggs Institute (JBI) Checklist for case series with an overall judgement for their quality.*

| **Criteria** | **Chen et al. (2023)** | **Justification** | **Ge Zhi-Zheng et al. (2011)** | **Justification** |
| --- | --- | --- | --- | --- |
| Domain 1: Randomization | Low | The randomization was computer based, and it was well balanced | Some concerns | The randomization and concealment was unclear |
| Domain 2: Deviations | Low | Since it was a double blind study following the intention-to-treat analysis it had a low risk of possible deviations | Some concerns | Since it was an open label study and had possible deviations |
| Domain 3: Missing data | Low | The use of an ascertainment framework limits the missing data related to outcome. | Low | Most patients were followed for greater than 1 year for accurate outcomes. |
| Domain 4: Outcome measurement | Low | The outcomes were prespecified such as bleeding, transfusion and hemoglobin levels. | Low | The outcomes (hemoglobin, bleeding episodes and transfusions) were measured objectively |
| Domain 5: Selection of results | Low | Intention-to-treat analysis used, a prespecified statistical analysis plan was used. | Some concerns | The protocol or statistical analysis plan used was not clear |
| Overall risk of bias | **Low** | Minimum bias was reported as per our understanding, as an appropriate randomization process, deviations, outcome measurements and technical analysis was used. | **Some concerns** | It was an open label study with a lack of blinding that can lead to potential deviations, and there was no documented protocol. |

**Supplementary Table S2**

*Detailed risk of bias assessment for randomized controlled trials Chen et al. (2023) and Ge et al. (2011) using the Cochrane risk of bias version 2 tool****.*** *The table shows domain-level judgments and justifications alongside an overall judgement for the studies. All the signalling questions with justifications are available in Supplementary file S1 an excel sheet.*

| **Criteria** | **Response for Garrido et al. (2012)** | **Justification** |
| --- | --- | --- |
| Domain 1: Bias due to confounding | Moderate risk | There was no randomization or control groups, the participants had multiple failed previous therapies. There was no statistical control for confounders. |
| Domain 2: Bias in selection of participants into the study | Moderate risk | There was a clear inclusion criteria of patients with recurrent bleeding due to angiodysplasia that is refractory to other treatments. However, the sample size is small leading to potential bias. |
| Domain 3: Bias in classification of interventions | Low risk | All patients received the same treatment (thalidomide 200mg/day). |
| Domain 4: Bias due to deviations from intended interventions | Low risk | Patients adhered to treatment guidelines there was no evidence of any deviations. |
| Domain 5: Bias due to missing data | Low risk | All the patients underwent follow-up, no evidence of any missing data. |
| Domain 6: Bias in measurement of outcomes | Moderate risk | Outcomes were mainly objective such as hemoglobin levels, transfusion requirements and bleeding episodes. However, lack of blinding poses a risk for measurement of outcomes. |
| Domain 7: Bias in selection of the reported result | Moderate risk | The protocol used was mentioned. The only reported result was vaguely mentioned as an improvement in bleeding only. |
| Overall ROBINS-I Judgment | **Moderate risk of bias** | Overall, it had proper interventions and no bias in inclusion. However, lack of protocol, confounding and a small sample size make it have a moderate risk of bias. |

**Supplementary Table S3**

*The table includes a risk of bias assessment for the observational study by Garrido et al. (2012) using the risk of bias in Non-Randomized Studies of Intervention (ROBINS-I) tool. The judgements alongside justifications are presented for all 7 domains with an overall risk of bias judgement.*

***Search String:***

***Cinahl: 14 citations***

***Search string:*** *(GI Bleed OR Gastrointestinal Hemorrhage) AND (Thalidomide OR Octreotide) AND*

*Angiodysplasia*

***Search done:*** *1/25/24*

***Scopus: 464 Citations***

***Search string:*** *(angiodysplasia ) AND ( ( thalidomide ) OR ( octreotide ) ) AND ( ( "gastrointestinal hemorrhage" ) OR ( "GI Bleed" ) )*

***Detail:*** *(angiodysplasia) AND ((thalidomide) OR (octreotide)) AND (("gastrointestinal hemorrhage") OR ("GI Bleed")) AND ( LIMIT-TO ( EXACTKEYWORD,"Angiodysplasia" ) OR LIMIT-TO ( EXACTKEYWORD,"Gastrointestinal Hemorrhage" ) OR LIMIT-TO ( EXACTKEYWORD,"Human" ) OR LIMIT-TO ( EXACTKEYWORD,"Humans" ) OR LIMIT-TO ( EXACTKEYWORD,"Article" ) OR LIMIT-TO ( EXACTKEYWORD,"Octreotide" ) OR LIMIT-TO ( EXACTKEYWORD,"Review" ) OR LIMIT-TO ( EXACTKEYWORD,"Thalidomide" ) OR LIMIT-TO ( EXACTKEYWORD,"Case Report" ) OR LIMIT-TO ( EXACTKEYWORD,"Gastrointestinal Bleeding" ) OR LIMIT-TO ( EXACTKEYWORD,"Bleeding" ) OR LIMIT-TO ( EXACTKEYWORD,"Controlled Study" ) OR LIMIT-TO ( EXACTKEYWORD,"Clinical Trial" ) OR LIMIT-TO ( EXACTKEYWORD,"Treatment Outcome" ) ) AND ( EXCLUDE ( SUBJAREA,"VETE" ) ) AND ( LIMIT-TO ( DOCTYPE,"ar" ) OR LIMIT-TO ( DOCTYPE,"re" ) OR LIMIT-TO ( DOCTYPE,"cp" ) OR LIMIT-TO ( DOCTYPE,"ed" ) )*

***Search done:*** *1/25/24*
